# Thoracoscopic resection of thoracic esophageal duplication cyst containing ectopic pancreatic tissue in adult

**DOI:** 10.1186/1749-8090-6-118

**Published:** 2011-09-25

**Authors:** Masashi Takemura, Kayo Yoshida, Keiichirou Morimura

**Affiliations:** 1 Department of Upper Gastrointestinal Surgery, Hyogo College of Medicine, 1-1, Mukogawa-machi, Nishinomiya City, Hyogo, 663-8501, Japan; 2 Department of Gastrointestinal Surgery, Osaka City General Hospital, 2-13-22, Miyakojima hondori, Miyakojima, Osaka, 534-0021, Japan

**Keywords:** esophageal duplication cyst, thoracoscopic surgery, ectopic pancreas

## Abstract

Esophageal duplication cyst is a rare congenital anomaly. They can be associated with other congenital anomalies, such as spinal abnormalities, and tracheoesophageal fistulas. In adults, almost of the patients with esophageal duplication cyst is asymptomatic and accidentally diagnosed by chest X-ray or computed tomography. However, cysts may become symptomatic owing to complications such as esophageal stenosis, respiratory system compression, rupture, infarction, or malignancy. Complete surgical resection is the standard treatment even in patients with asymptmatic cysts. Traditional approach for resection is via thoracotomy. But, the thoracoscopic approach makes more indicate for mediastinal diseases, because of minimally invasive for patients. We describe a case with esophageal duplication cyst, which contained the ectopic pancreatic tissue in the solid portion, resected under the thoracoscopic approach in adult.

## Background

In adults, the patients with esophageal duplication cysts are asymptomatic and accidentally diagnosed on chest X-ray photograph or computed tomography. Cysts may become symptomatic owing to various complications such as esophageal stenosis, respiratory system compression, rupture, infarction, or malignancy [1-5]. Definitive treatment involves complete surgical resection of the cysts via thoracotomy, even in asymptomatic [6,7]. But, in recent years, the thoracoscopic approach makes more indicate for mediastinal diseases [8,9].

In this report, we describe a case of esophageal duplication cyst, which contained the ectopic pancreatic tissue in the solid portion of cyst, was resected under the thoracoscopic approach in a young adult.

## Case presentation

**Figure 1:**
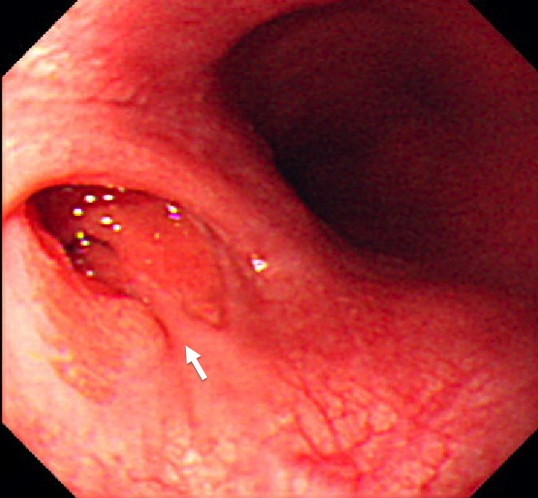
**Gastrointestinal endoscopy showed the esophageal diverticulum in the left side of middle thoracic esophagus, covered with columner epithlium (arrow)**.

**Figure 2:**
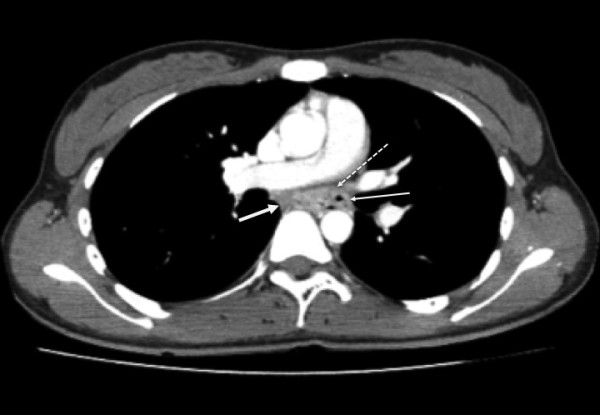
**Chest computed tomography showed a mediastinal mass lesion at caudal side of tracheal carina approximately 3 cm diameter**. The lesion contained partially air density part (thin solid line) and solid portion (bold solid line). Dotted line showed esophageal lumen.

A 21-year-old woman with history of repeated chest pain was admitted to our hospital. She had been initially diagnosed mediastinal abscess due to rupture of esophageal diverticulum at another facility. Blood examination showed leucocytosis (12430/mm^3^), but normal level of C-reactive protein. Chest x-ray photograph revealed no sign of mediastinal mass and pleural effusion. The bilateral lung fields were apparently normal. The chest vertebral bodies and intervertebral disc spaces have unremarkable changes. An upper gastrointestinal endoscopy showed the esophageal diverticulum lined columnar epithelium at left side of middle thoracic esophagus (Figure 1). Chest computed tomography showed a meditational mass at caudal side of tracheal carina at the left side of middle thoracic esophagus, and maximum diameter of approximately 3 cm. The mass lesion have thin wall and contained partially air density part and solid portion (Figure 2). The patient was diagnosed as having a mediastinal abscess due to perforation of esophageal diverticulum from these findings.

**Figure 3:**
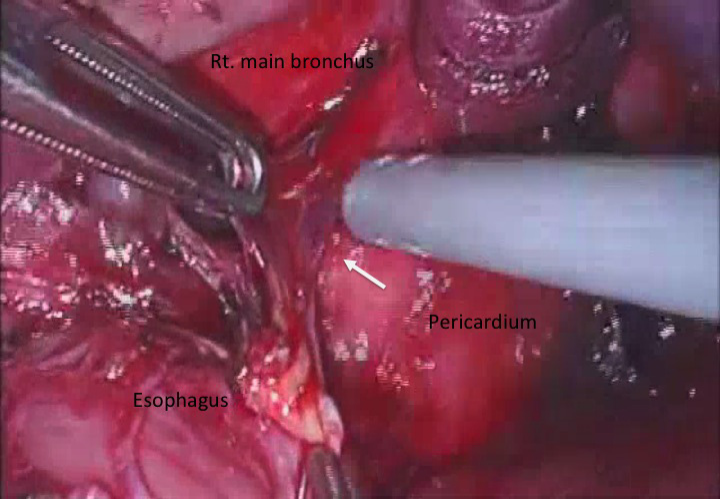
**The middle thoracic esophagus was isolated from surrounding organs**. The fibrous changes due to repeated inflammation were noted (arrow).

**Figure 4:**
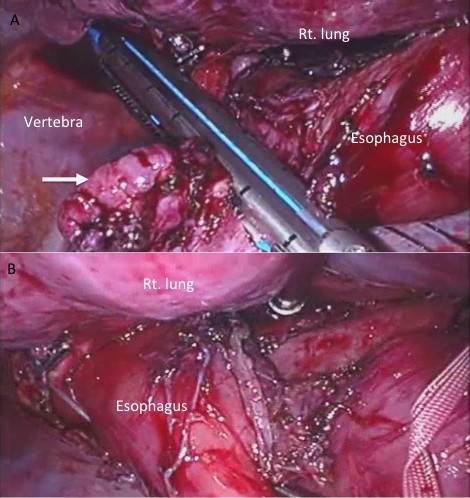
**The esophageal lesion was resected using linea stapler**. A; The lesion was resected along the major axis of the esophagus (arrow). B; The esophagus was not narrow after resected the lesion.

Surgery was carried out via right thoracoscopic approach. The double lumen endotracheal tube was used for deflates the right lung. The arch of azygos vein was ligated and cutted. The middle thoracic esophagus was isolated from pericardium and carina at ventral side (Figure 3). The fibrous change due to repeated inflammation of adjacent structures was noted. Bilateral vegal nerve identified and preserved. The operation was proceeding with the aid of endoscope in the esophagus, checked for investigation the air insufflation intraoperatively. After secured the middle thoracic esophagus, the lesion was resected using linea stapler (Figure 4A, B).

**Figure 5:**
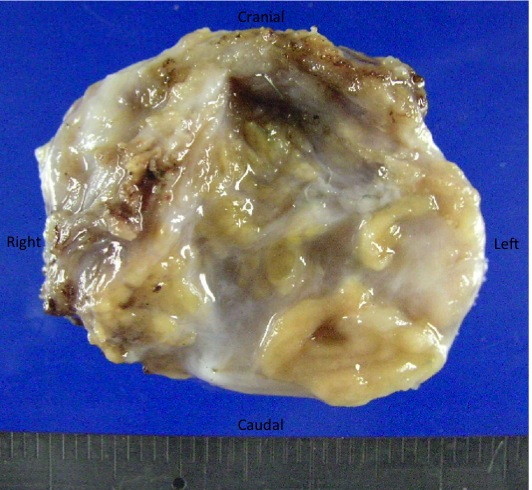
**Resected specimen was 3.5 × 3.5 cm in diameter**.

**Figure 6:**
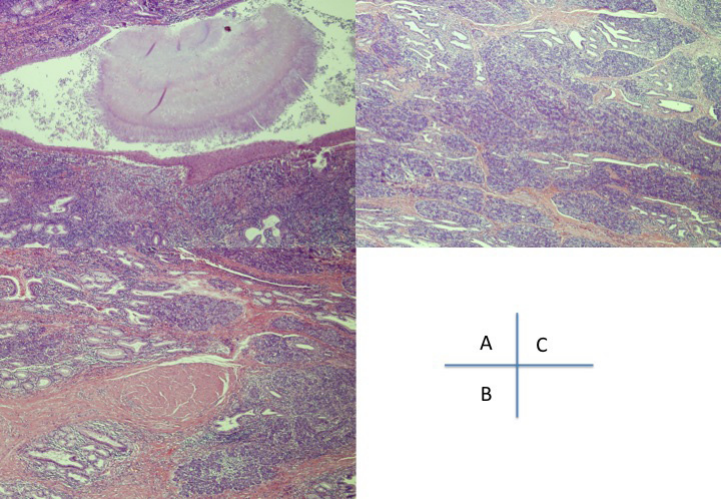
**Pathological findings of the resected specimen**. (H.E. × 40)A A; The cystic part of the lesion lined by squamous epithelium, columner or simple cuboid epithelium complicated actinomycetic granule. B; The cyst covered by smooth muscle layer. C; The solid portion of the lesion contained multiple solid foci of pancreatic tissue.

Resected specimen showed 3.5 × 3.5 cm cystic tumor with solid portion (Figure 5). Pathologically, the resected specimen was composed of cystic part and solid portion. The cystic part of the lesion lined by squamous epithelium, columner or simple cuboid epithelium complicated with actinomycetic granule. The cyst covered by smooth muscle layer (Figure 6A, B). The solid portion consisted of admixture of glands of fundic types. In addition, multiple solid foci of pancreatic tissue were scattered (Figure 6C). This lesion was diagnosed as esophageal duplication cyst from these findings. Post operative course was satisfactory, and the patient was discharged from our hospital at 12 days postoperatively. She was symtoms-free at 9 months from operation.

## Discussion

The esophageal duplication cysts estimated at 20% of alimentary tract duplications, make it the second most common site [1,2]. In adults, esophageal duplication cysts usually are diagnosed incidentally because of most cases has asymptomatic. However, they become symptomatic when complications occur, such as obstruction, rupture, hemorrhage, infection and rarely developed malignancies [3-5]. The esophageal duplication cysts arise from the foregut embryologically. Lower respiratory system, esophagus, stomach, hepatobiliary system, and pancreas developed from foregut. So, the esophageal duplication cysts may contain these components pathologically. Actually, ectopic gastric mucosa in esophageal duplication cysts was found in 43% [2]. However, esophageal duplication cysts with pancreas components are rare [2]. Qazi et al [10] reported the resected case with esophageal duplication cyst complained the recurrent retrosternal pain. In this case, the cyst contained pancreatic components in the solid portion pathologically. They suggested that the destructive action of pancreatic enzyme contributes to the patient symptoms. Our case demonstrated recurrent episode of chest pain, too. The secretory actions of pancreatic tissue might have related to her symptoms.

Definitive treatment of esophageal duplication cyst is complete surgical resection. Conventional approach is under thoracotomy or laparotmy [1,6]. Moreover, recent advances in minimally invasive surgery have led to less traumatic approach for the treatment of benign mediastinal lesions. Actually, many cases with esophageal duplication cysts treated by thoracoscopic technique have been reported [7,9]. The points that should be careful for resection of the esophageal duplication cyst were 1) preserving the muscle layer, 2) both vegal nerves should be identified and preserved, 3) mucosal integrity should be checked intraoperatively by air insufflation [7]. A thoracoscopic approach can contribution to a precise resection of the cysts as open thoracotomy dose.

## Conclusions

In adults, almost of the patients with esophageal duplication cyst is asymptomatic and accidentally diagnosed by chest X-ray or computed tomography. However, cysts may become symptomatic owing to complications such as esophageal stenosis, respiratory system compression, rupture, infarction, or malignancy. In our case, the symptoms may relate to the pancreatic component in the cyst. Even in such cases, thoracoscopic approach was safety and useful procedure.

## Consent

Written informed consent was obtained from the patient for publication of this case report and any accompanying images. A copy of the written consent is available for review by the Editor-in Chief of this journal.

## Competing interests

The authors declare that they have no competing interests.

## Authors' contributions

MT drafted and finalized the manuscript, prepared the figures. KY reviewed the manuscript and prepare the figures. KM prepare the manuscript and performed gastroendoscopy.

All authors read and approved the final manuscript.
